# Hospital clinicians’ perceptions and experiences of care pathways for chronic limb-threatening ischaemia: a qualitative study

**DOI:** 10.1186/s13047-023-00664-6

**Published:** 2023-09-19

**Authors:** Eleanor Atkins, Ian Kellar, Panagiota Birmpili, Jonathan R. Boyle, Arun D. Pherwani, Ian Chetter, David A. Cromwell

**Affiliations:** 1 Clinical Effectiveness Unit, Royal College of Surgeons of England, 38-43 Lincoln’s Inn Fields, Holborn, London, WC2A 3PE UK; 2 Hull York Medical School, Hull, UK; 3 University of Sheffield, Sheffield, UK; 4 Department of Vascular Surgery, Cambridge University Hospitals, Cambridge, UK; 5 Staffordshire & South Cheshire Vascular Network, Royal Stoke University Hospital, Stoke-On-Trent, UK; 6 Department of Health Services Research and Policy, London School of Hygiene and Tropical Medicine, London, UK

**Keywords:** Vascular surgery, Chronic limb threatening ischaemia, Referral and consultation

## Abstract

**Background:**

Chronic limb-threatening ischaemia (CLTI) is a condition associated with significant risks of lower limb loss and mortality, which increase with delays in management. Guidance recommends urgent referral and assessment, but delays are evident at every stage of the CLTI patient pathway. This study uses qualitative methods to explore hospital clinicians’ experiences and perceptions of the existing CLTI pathway.

**Methods:**

A qualitative interview study was conducted. Semi-structured interviews were undertaken with 13 clinicians involved in the assessment of patients referred to hospital with suspected CLTI, identified via purposive sampling from English vascular surgery units. Clinicians included podiatrists, vascular specialist nurses and doctors. Reflexive thematic analysis was performed on the data from a critical realist position.

**Results:**

The need for speed was the single overarching theme identified. Four linked underlying themes were also identified;

1. Vascular surgery as the poor relation (compared to cancer and other specialties), with a sub-theme of CLTI being a challenging diagnosis.

2. Some patients are more equal than others, with sub-themes of diabetes vs. non-diabetes, hub vs. spoke and frailty vs. non-frail.

3. Life in the National Health Service (NHS) is tough, with sub-themes of lack of resource and we’re all under pressure.

4. Non-surgeons can help.

**Conclusions:**

The underlying themes generated from the rich interview data describe barriers to timely referral, assessment and management of CLTI, as well as the utility of non-surgical roles such as podiatrists and vascular specialist nurses as a potential solution for delays. The overarching theme of the need for speed highlights the meaning given to adverse consequences of delays in management of CLTI by clinicians involved in its assessment. Future improvement projects aimed at the CLTI pathway should take these findings into account.

**Supplementary Information:**

The online version contains supplementary material available at 10.1186/s13047-023-00664-6.

## Background

Chronic limb-threatening ischaemia (CLTI) is the end stage of peripheral arterial disease (PAD) and is associated with significant mortality and morbidity [1]. The lack of blood flow to the lower limbs causes pain in the feet at rest or at night, and / or tissue loss such as gangrene or non-healing ulceration. Tissue loss can be anywhere in the lower limb, but is most often seen on the foot. Revascularisation in CLTI, carried out by vascular surgeons, is associated with improved mortality and limb salvage outcomes [2].

In England, vascular services are organised into networks. These comprise one hub, or arterial centre, which carries inpatient arterial surgery, including emergency vascular surgery, and a number of spoke hospitals, or non-arterial centres, which have limited vascular surgery presence [3]. The established minimum population for one vascular network is 800,000 [3]. Each network should have a documented patient pathway in place for the timely management of CLTI, including arrangement for urgent transfer [4].

There are delays at every stage of the patient pathway in CLTI, from identification to revascularisation [5]. Delays in revascularisation are associated with increased mortality and limb loss [6]. Following variation identified in the timing of revascularisation by the Getting It Right First Time programme [7], the Vascular Society of Great Britain and Ireland (VSGBI) released guidance stating primary care clinicians should be referring patients with suspected CLTI to vascular surgery services on the same day they see the patient. Vascular surgeons were also given challenging targets for time-to-revascularisation from referral [8]. This applies to all patients covered by the network, whether they live near a hub or a spoke.

There has been little research on factors affecting the processes occurring prior to expert assessment for suspected CLTI, but we know there are missed opportunities in primary care to refer patients with CLTI, and barriers to patients accessing appropriate care [9, 10]. Nickinson et al. suggested patient factors can affect the timely recognition and referral of CLTI by primary care clinicians, including age and deprivation [9]. Lecouturier et al. identified factors affecting diagnosis and referral of PAD in primary care including a lack of awareness of guidelines, dependence on ankle-brachial pressure index (ABPI) and patient delay in presentation [11].

Further work has been called for to investigate factors affecting timely referral to secondary care for patients with suspected CLTI [9]. The aim of this study was to explore the experiences and perceptions of clinicians who are involved in the triage and assessment of patients with suspected CLTI of the processes currently in place, in order to inform future improvement projects. Here, we report findings from thematic analysis of qualitative interviews with these clinicians.

## Methods

In order to fulfil the aim of our research, a qualitative interview study was conducted. Qualitative research enables researchers to understand how people view the world around them [12]. Design was pragmatic, according to resource available, whilst ensuring conceptual coherence with the research questions. We sought to investigate participants’ individual experiences and perceptions, and define any common meaning, understanding the contextual location within the structures participants are working in. The analysis was approached from a critical realist position, where multiple experiences and perceptions of a single reality exist, combining ontological realism with epistemological relativism. Critical realism understands knowledge and experience to be articulated through language [13] and consequently mostly semantic data were coded. The main criteria for coding and theme development was meaning, as opposed to recurrence, in keeping with a “big Q” qualitative paradigm, with a fully qualitative approach [14]. This avoided a lean towards positivism, or searching for a single truth in our data. Reflexive thematic analysis was used, a method that entails identification, analysis and reporting of patterns within the data [15]. It was initially described by Braun and Clarke in 2006 [16], and acknowledges that the researcher is part of the world they wish to understand. Its flexibility allowed us to inductively develop an analysis according to our critical realist position. Reflexive thematic analysis is a method considered useful in under-researched areas such as this one, and it can produce analyses suited to informing policy change [16]. The consolidated criteria for reporting qualitative research (COREQ) has guided the reporting of this study [17] (Additional file 1).

Full ethical approval was granted by the Hull York Medical School Ethics Committee (ref. 21/22 32).

### Identification and recruitment of participants

This study followed a process mapping study [18], which involved detailed interviews around processes in place for referrals with suspected CLTI in 12 English hospitals with vascular surgery services.

Initial analysis in the process mapping study categorised pathways into three themes according to staff group involvement. Purposive sampling was used to identify clinicians for interview, with four clinicians being recruited from each theme, ensuring all staff groups were captured (Table 1). This ensured maximum variation of experiences with referral pathways in our sample.

**Table 1:** Sampling grid for vascular clinicians

	Vascular surgeon	Podiatrist	Vascular specialist nurse
Surgeon-led pathway	2	1	1
Podiatry-led pathway	1	2	1
Nurse-led pathway	1	1	2

The chosen number of participants was informed by Guest et al.’s recommendations for qualitative interview studies in relatively homogenous groups, following an experiment in data saturation where 12 interviews were found sufficient to understand common perceptions and experiences [19]. Reflection on the content of our dataset during the familiarisation process found the 13 interviews to contain adequate richness to fulfil our research aims, as per the concept of information power [20].

Selection of potential participants was based on their previous engagement with the process mapping project, and their stated willingness to be involved with ongoing work. Recruitment of a broad range of hospitals was also prioritised. The sole inclusion criterion was that the clinician had participated in the process mapping project as an interviewee. There were no exclusion criteria.

### Information and consent

Potential participants were invited to be part of the study over email, with a brief explanation of the planned project and a Participant Information Sheet (PIS) attached. Opportunity was given for further explanation and any questions to answered, and once the participant was ready, a mutually suitable time for online interview was agreed. Consent was confirmed verbally both before and after the online interview, and a signed consent form was received from the participant.

### Interviews

Semi-structured interviews were carried out by EA, a female vascular surgeon working towards a postgraduate qualification. Whilst she has had no formal interviewing training, much of the clinical work of the surgeon involves similar techniques [21].

Interviews were carried out online, using Microsoft Teams. Non-participants were not present. A pre-piloted topic guide was used as a framework for the interviews, which was iteratively altered as the study progressed (Additional file 2). This posed open questions about different stages of the patient’s pathway where delays were possible. Prompts were used, such as, “can you tell me more about that?” when further details were required.

As a vascular surgeon, EA had pre-existing assumptions and situated knowledge around the research question. Care was taken during the interviews to remain neutral and not express opinions throughout, or to lead the participant. Reflexive thematic analysis acknowledges the researcher’s subjectivity, and uses it to inform analysis [15]. A reflexive diary was kept throughout the process, and individual reflections written after each interview, including information on how pre-existing assumptions were challenged. Data coding and theme generation were informed by regular reflection, seeking to understand the researcher’s subjectivity and ensure all meaningful data was coded. This led to a complex, nuanced analysis of the data.

### Analysis

Each interview was audio and video recorded and Microsoft Teams software used to carry out an initial transcription. This transcription was edited according to the audio recording until it was verbatim, then anonymised. According to Braun and Clarke’s six phases of thematic analysis, immersion in the data took place both with the audio recordings and the transcripts, with initial notewriting of questions, observations and interpretations. Familiarisation began after completion of all the interviews. Formal inductive coding was carried out by EA using Nvivo software, followed by two cycles of re-coding. Both semantic and latent coding took place. Candidate themes were generated initially, then developed and revised following discussion with the wider author team and re-engagement with the original data. Themes were then refined, defined and named. The connections between themes were discussed and mapped visually.

## Results

Twelve participants were approached for inclusion in the study, and all accepted the invitation. None dropped out or rescinded consent at a later stage. Four vascular specialist nurses, four podiatrists and four vascular surgeons from 10 of the 12 hospitals previously involved in the process mapping were interviewed (Table 1). One further interview was included, from a medical clinician involved in the assessment process of one of the surgeon-led pathways, with whom the topic guide had been piloted. The interviews lasted between 30 and 64 min. All participants were known to EA prior to the study commencement. Participants knew EA’s background as a vascular surgeon, and understood the purpose of the research. Reflection on the content of our dataset during the familiarisation process found the 13 interviews to contain adequate richness to fulfil our research aims, as per the concept of information power [20].

**Fig. 1:**
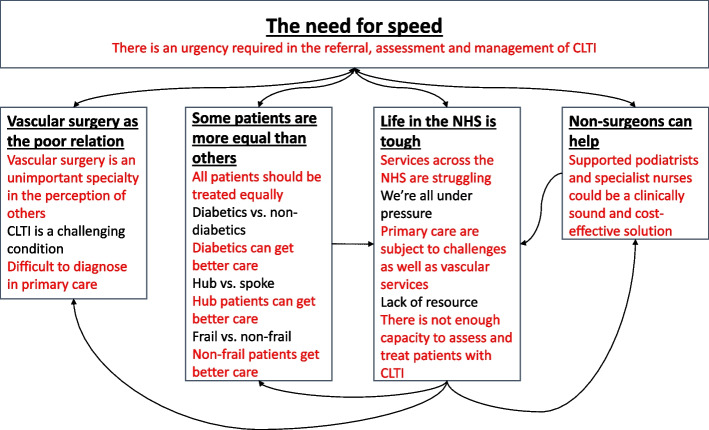
Themes and sub-themes

Four key themes were developed: vascular surgery as the poor relation; some patients are more equal than others; life in the NHS is tough; and non-surgeons can help. These will be discussed in turn, with reference to sub-themes. They are linked by one overarching theme, the need for speed (Fig. 1).

### Theme 1: vascular surgery as the poor relation

The theme “vascular surgery as the poor relation” reflects the core idea expressed across the dataset that vascular surgery, the specialty which deals with CLTI, is an unimportant specialty in the perception of others. This was reflected in a perceived lack of awareness of CLTI, across patients, primary care clinicians and hospital management.

“I think peripheral vascular disease has always been one of those things that hasn't really been studied, particularly through medical training. It's one of the things that's touched briefly on, but not really in depth. And it's, I think it's just lack of awareness really.” Vascular specialist nurse, podiatry-led pathway.

“Patients describe things like, a bit of a scab or, I banged my toe, or, it’s a bit weepy round my nail. Me and you would describe that as gangrene. And I think that the normal, generalised population haven’t got the words to be able to articulate what’s going on with their foot, so that it equals critical limb ischaemia in the clinician’s mind.” Vascular specialist nurse, nurse-led pathway.

This unimportance was demonstrated by participants in comparison to other conditions, including cancer, stroke and heart attack.

“But I do think that, you know, you get all this education for strokes and things like that, but is it as well thought out of, like publicized in the wider public about recognizing the symptoms of CLTI, than it is, you know, these like things like stroke and heart attack and things like that.” Podiatrist, podiatry-led pathway.

CLTI was described as a challenging condition – more challenging than others, which is a sub-theme within this idea.

“I think that it's very difficult to identify critical limb ischaemia. I think that our patients are very complex. They are presenting with neuropathic pain and vascular pain, and sometimes that can be difficult to differentiate.” Podiatrist, surgeon-led pathway.

The lack of awareness of CLTI perceived by participants was deemed responsible for delays. Participants described this in the context of poor referral quality, lack of a shared language between patients, primary care clinicians and secondary care clinicians, and in engagement of management to facilitate urgent treatment pathways. Vascular surgery’s status as the poor relation was expressed as something that could be changed, with national campaigns, education and improved relationships between primary care and clinicians who assess CLTI recommended.

#### Sub-theme: CLTI is a challenging condition

Participants presented the difficulty of making a diagnosis of suspected CLTI in primary care was as three-fold: a challenging group of patients who suffer from this condition; characteristics of the condition itself; and the wide range of primary care clinicians who it may present to.

“I think because the symptoms of rest pain, or perceived pain in in the extremity can overlap with lots of fairly benign conditions, and therefore they probably feel a little bit nervous about referring something urgent that could be something very benign.” Surgeon, podiatry-led pathway.

“I think with CLTI, though, and the variety of symptoms you've got, you're not necessarily gonna end up with a nurse. You could end up in a GP [general practitioner], in the podiatrist, in the diabetic centre, within the nursing service because—is it pain on your foot? Is it? Is it a scab? Is it a toenail? Is it an infection? And there's so many ways that it can be badged initially, that many, many people could see it in terms of that first recognition.” Vascular specialist nurse, nurse-led pathway.

### Theme 2: Some patients are more equal than others

Inequalities in the pathway from first symptom to assessment were described within three sub-themes. They were presented throughout as undesirable – it was clear that participants thought that all patients with suspected CLTI should ideally be treated equally, with no discrimination against or in favour of a specific group of patients.

“…get rid of the diabetic foot clinic and just have a lower extremity wound clinic or something. You know, the limb salvage approach where, because it's the same, it's the same pathology. It's just some arbitrary cut-off which has been put there… It probably made sense 20 years ago and it doesn't anymore. And I think that is probably the direction we should be heading.” Medical clinician, surgeon-led pathway.

Each of the three sub-themes represents a different source of inequality.

#### Sub-theme 1: People with diabetes vs. people without diabetes

People with diabetes were viewed by participants as receiving better care than people without diabetes, with increased awareness of symptoms in patients and primary care clinicians, the availability of alternative (better) pathways into assessment by an appropriate clinician and services for people with diabetes being prioritised with funding.

“And I don't think that's the case with diabetes, because diabetic patients, patients with diabetes have a bit more general education, because it's more of a progressive illness over time, so they get regular checks and regular education. So I think they're probably a bit more switched on about attending when they develop…” Surgeon, nurse-led pathway.

“I think because the diabetic foot service is so good, they're very keen to flag patients that we need to see. And in some ways they probably get a better service, because they've been managed, you know, from early and I think those patients have rapid access to the podiatrists anyway. So a lot of those patients will know if they get a foot wound, they fall and they come and see podiatry, and podiatry will then flag it. So I think those patients probably do quite well actually out of their service.” Surgeon, surgeon-led pathway.

There was a sense of disappointment and unfairness, that people should be treated differently due to the presence of a comorbidity.

#### Sub-theme 2: Hub vs. spoke

Participants saw reconfiguration of vascular services into a network model as having led to difficulties in accessing care for patients, and reconfiguration has been seen as leading to substandard care for some of the populations covered by the network.

“Sadly, I think the, one of the downsides personally in my view would be with the, sort of, centralization of services, is that we've taken away the expertise out of the spoke hospitals, and so, many people with foot problems are managed by clinicians who have no experience.” Surgeon, surgeon-led pathway.

“I think the spoke patients have more delay to being seen. And that is because in [Unit] we have, you know, four times a week CLI clinic, whereas we don't have that in any of the other spokes.” Surgeon, surgeon-led pathway.

#### Sub theme 3: frail vs. non-frail

Patient frailty and the presence of comorbidities were said by participants to affect options for assessment negatively, particularly those patients who required hospital transport, or those who were unable or unwilling to travel long distances. More complex assessment processes were required in these cases, which took time and led to frustration.

“Now we know that not every ambulatory patient, or not every patient with CLTI is ambulatory and equally just because the patient is bedbound with CLTI, does not mean that they shouldn't be reviewed. But we do have a massive issue with being able to get these patients into hospital because we – ED [emergency department] is not an appropriate route for them, they can't come to the surgical triage unit because they come bedbound, they're hoisted and there isn't space or staff to care for them.” Vascular specialist nurse, nurse-led pathway.

### Theme 3: life in the NHS is tough

This theme comprises two sub-themes. Overall, the theme notes that services in the NHS are struggling. Clinicians felt they could not offer optimum care to patients, or perceived that other clinicians were prevented from offering optimum care by constraints external to their individual clinical practice.

“I think one of the major barriers, especially for the community nursing team is just staffing turnover. So they get, they just seem to have a massive turnover of band five and six staff that just constantly move on. Recruitment battles is a big thing, so everything becomes so much more fraught.” Podiatrist, nurse-led pathway.

The first sub-theme considers the pressure across all services. In the second sub-theme, participants noted the lack of resource present for improvement, or indeed to provide an adequate service for patients with CLTI.

#### Sub-theme 1: We’re all under pressure

Participants reported working in a pressurised environment in the hospital. An increased demand for vascular surgery care was described, with challenges arising from inadequate staffing, the Covid-19 pandemic and competing priorities.

“So there's an issue from a staffing point of view as well is that we've had a significant increase in the number of patients that we receive into the service, and yet our staffing and our infrastructure remains exactly the same as it was five years ago.” Vascular specialist nurse, nurse-led pathway.

Participants had an appreciation that these pressures extend into primary care and affect primary care clinicians, as well as patient access to primary care clinicians.

“And again, like, I appreciate what it's like for clinicians in the community, and the time constraints, busy clinics, patient after patient coming in.” Podiatrist, podiatry-led pathway

#### Sub-theme 2: lack of resource

The idea that there was a lack of resource was strongly expressed throughout the interviews. This was detailed both in terms of the capacity to assess patients and treat them once the diagnosis of CLTI had been made.

“So we're capturing the patients in the much earlier stages, but actually getting them that angioplasty, or that surgical intervention has – it’s sort of highlighted that there's a bit of a delay. And certainly our consultant diabetologist, on Friday, I said, you know, we've seen this patient, he's gonna have—he's had his duplex scan, he's gonna have an angioplasty and there was no like, great we've done that in 24 h. It was like yes, but how long is he gonna wait for an angioplasty, you know what I mean?” Vascular specialist nurse, nurse-led pathway.

Improvement in the current service was perceived to require additional resource, or lead to the worsening of care for other patients. The lack of resource for timely intervention for CLTI once assessed and diagnosed was seen as a barrier to encouraging improvements in timely referral from primary care clinicians.

### Theme 4: Non-surgeons can help

The final theme communicates a potential solution to the timely assessment of patients with suspected CLTI. Participants perceived non-surgeons involved in care pathways, such as podiatrists and vascular specialist nurses, to be key facilitators of the processes in place for assessment of patients referred with suspected CLTI.

“However, podiatry are very good at triage and stuff, so if they're not sure about presentations or what exactly is going on, I know that they will see their patients regardless and pass on quickly if needed. So they are very good at picking up stuff.” Vascular specialist nurse, podiatry-led pathway.

Participants said the involvement of non-surgeons, with support from vascular surgeons, was clinically sound and more cost-effective than the use of surgeon time, as well as, in the case of vascular specialist nurses, adding holistically to patient care.

“It's probably a better use of [vascular specialist nurse’s] time rather than our time, I suspect, if you're looking at the, you know, cost benefit.” Surgeon, nurse-led pathway

“Maybe it's that patients feel more comfortable with nurses. I think it's something about the caring role that nurses do that, I think, patients feel more comfortable telling nurses things they wouldn't necessarily tell doctors.” Vascular specialist nurse, nurse-led pathway.

Participants described increased responsibility within roles as enabling clinicians to work at the top of their game – including the vascular surgeons non-surgeons were seen to be protecting from the work of assessing patients.

“I also think as well, we have to ration consultants to be where they need to be. So to me, a consultant needs to be on call. They need to be responding to the trauma bleep. Or they need to be operating because they are all the things that only a surgeon can do.” Vascular specialist nurse, nurse-led pathway.

The importance of good administration support in enabling timely assessment was also a clear idea within this theme.

### Overarching theme: The need for speed

The urgency required in the management of CLTI was an overarching theme, emphasised throughout the interviews and linking all four themes described. The perceived unimportance of CLTI represents a cause of delay according to the participants, whether because patients don’t present with symptoms they put down to other causes, primary care clinicians don’t recognise the symptoms as being due to CLTI, or vascular disease not being prioritised relative to other conditions.

“So I think GPs are very aware of all the two week cancer pathways. I don't think they're aware of the CLTI world. And I think that's very difficult as to how to tap into that.” Surgeon, surgeon-led pathway.

“Again, we see evidence of this all the time where GPs haven't picked up on this. They don't realize the repercussions and people have come in with late presentations, and obviously ultimately lost limbs.” Vascular specialist nurse, podiatry-led pathway.

Participants felt that inequalities can limit the speed at which some patients are assessed, and the pressure on services and lack of resource can explain delays in recognition, assessment and management of CLTI.

“If I have a diabetic patient in the same situation, I could get them to see vascular on Thursday. So there is quite a difference between, say, diabetes and non-diabetes. And having a diabetes label, certainly, you know, things move along a lot quicker, or have more access to services quicker.” Podiatrist, nurse-led pathway.

Delays in the pathway were thought to lead to adverse outcomes, and the importance of a timely process from first symptom to assessment by an appropriate clinician was evident throughout the dataset.

“One of the things I think that should happen is that the sooner we see someone and get a diagnosis about why they have a foot problem and what we're going to do about it the better.” Medical clinician, surgeon-led pathway.

“The big delays for us, as soon as the patient gets to us, is now cross-sectional imaging. And we've got huge delays, and that's a post-Covid thing. So an urgent scan now with us will take at least 6 to 8 weeks.” Surgeon, podiatry-led pathway.

## Discussion

This qualitative study explored the perceptions and experiences of hospital clinicians involved in the processes in place for patients with suspected CLTI. Our finding of the overarching need for speed in this process is supported by national and international guidance documents where urgent, prompt or early referral in the case of suspected CLTI is recommended [8, 22–24]. There is, however a perception amongst clinicians that this importance is not shared by referring clinicians in primary care, and vascular disease is seen as “lesser than” other conditions. This perception is supported by a previous survey study, which have found discrepancy in mortality perceptions between PAD and cancer [25]. Education and increasing of awareness has been suggested as a solution, for both clinicians and patients, and a survey study of registered podiatrists has indicated a need for education on assessment and referral of peripheral vascular disease [26]. Other studies using qualitative methodologies have investigated stakeholders’ experiences with referrals in different specialties, and found similar issues with referrer awareness and patient understanding affecting these processes [27, 28].

There was recognition that the whole of the NHS is under significant pressure, and is struggling with lack of resource. The King’s Fund has described a worsening workforce crisis in the NHS [29] and the Health Foundation report that people are living more years in poor health, life expectancy has stopped rising and inequalities are widening [30]. Funding of the NHS has failed to align with demand for services in the context of growing staff shortages [31], and it is clear that the hospital clinicians interviewed not only feel that pressure, but are aware that it extends to primary care as well.

Inequalities have been considered as a factor affecting referrals previously in terms of patient age and deprivation, both of which contribute to frailty [9]. Whilst major amputation rates have fallen in England, the rate of decrease was half as fast in people with diabetes than people without diabetes [32]. The emergence of multi-disciplinary diabetic foot teams (MDFTs), which are recommended by the National Institute of Health and Care Excellence (NICE) alongside clear timelines and foot care structures will likely have contributed to this decrease [33]. The provision of similar foot services for people without diabetes is much less widespread [34]. Vascular services have been reconfigured over the past decade into a hub and spoke model, leading to geographical changes in vascular presence across networks. Li et al. found that mortality and limb salvage outcomes for patients with CLTI who are referred to spoke hospitals are worse compared to those who present to hub hospitals [6] and people with diabetes from more deprived areas are more likely to be discharged from secondary care with a diagnosis of PAD and / or CLTI compared to those from less deprived areas, highlighting the importance of acknowledging geography and deprivation when creating or altering care pathways for CLTI [35].

The use of non-surgeons in the process of referral and assessment of CLTI has been widely documented. The inter-disciplinary team approach has been recommended for many years in the diabetic foot [36], and has had marked success in reducing major limb amputation rates [37–39]. More recently this recommendation has been extended to all wounds and CLTI care, according to the “toe-and-flow” model [1, 40, 41], with corresponding success in the limited literature [34]. Podiatrists in Greater Manchester have developed a community-based gatekeeper service for patients with PAD, including CLTI, improving patient access to vascular assessment and protecting vascular surgeon time [42]. In Leicester, the Vascular Limb Salvage (VaLS) clinic, which is specialist nurse-led, is a model of care able to provide timely assessment of suspected CLTI, and reduce amputations [43]. The benefits of the involvement of these staff groups in the patient pathway are valued by vascular surgeons, and formalising these roles in a recommended model of care may be a potential solution to some of the barriers to timely assessment.

### Strengths and limitations

In this qualitative study, we have collected and analysed rich interview data in order to explore hospital clinicians’ experiences and perceptions of the care pathway for CLTI. This was an appropriate study design, given little pre-existing evidence on this topic. The participants were diverse with regards to role, geography and process, which maximises potential for transferability of our results. However, the study included only hospital clinicians, and thus our results alone should not be used to implement changes to CLTI care pathways. There are other important stakeholders associated with this process whose views have not yet been explored. Further work should be carried out with patients and primary care clinicians to understand their experiences prior to considering any interventions on the referral process. The study included participants from ten vascular networks, representing less than 20% of the total number in England, and whilst the overall sample size was satisfactory according to data saturation and information power, there were small numbers of participants in each type of pathway from each staff group. This may mean that some perceptions existing in hospital clinicians have not been captured. Participants were also selected partly on the basis of engagement with a previous study, and this engaged cohort may have skewed attitudes towards CLTI pathways, therefore not representing hospital clinicians as a whole.

## Conclusions

This study indicates that clinicians involved in the assessment of suspected CLTI recognise the need for speed throughout the process to diagnosis. Further key themes generated from the data which prevent patients receiving timely care include inadequate resource and system pressures, lack of awareness in other clinicians and the public, and inequality across patient characteristics. A final theme, where non-surgeons can deliver appropriate care, has also been discussed as a potential solution. In addition to work with other stakeholders, the results of this study should be considered during the planning of any improvements relating to pathways from first symptom to expert assessment in patients with suspected CLTI. These could include increasing awareness of CLTI in primary care clinicians and the public, allocating adequate resource in order to alleviate pressure throughout the health system, and measures to promote equality across the patient cohort. The use of non-surgeons should be considered throughout the pathway.

### Supplementary Information

**Additional file 1.** COREQ checklist.

**Additional file 2.** Indicative interview topic guide.

## Data Availability

The datasets generated and/or analysed during the current study are not publicly available as participants were not consented for their data to be shared in this manner. Data are available from the corresponding author on reasonable request.

## Abbreviations

CLTI: Chronic limb threatening ischaemia
NHS: National Health Service
PAD: Peripheral arterial disease
VSGBI: Vascular Society of Great Britain and Ireland
ABPI: Ankle-brachial pressure index
COREQ: Consolidated criteria for reporting qualitative research
PIS: Participant Information Sheet
GP: General Practitioner
ED: Emergency Department
MDFTs: Multi-disciplinary diabetic foot teams
NICE: National Institute of Health and Care Excellence
VaLS: Vascular Limb Salvage
