# Supplementary material for: Hospital clinicians’ perceptions and experiences of care pathways for chronic limb-threatening ischaemia: a qualitative study
Source: J Foot Ankle Res. 2023 Sep 19;16:62. doi: 10.1186/s13047-023-00664-6 (PMC10507819; doi:10.1186/s13047-023-00664-6)
Supplement: Supplementary file 2 — Additional file 2. Indicative interview topic guide. [file 13047_2023_664_MOESM2_ESM.docx]

**Indicative interview topic guide: vascular surgery clinicians**

Introduction:

- My name
- My role
- Aims of interview
- Safety information

Consent

- Are you happy to proceed?

Start recording

Data on centre

- So first of all, I’ll ask you to confirm for me which centre you work in, and what your role is within the referral process

Primary care questions

- Before we go onto the specific process map for your area, I’ll ask some questions about primary care.
- Do you feel primary care clinicians have the ability to pick up on the symptoms / cues that should lead them to suspect CLTI?
  - If not, what are they lacking?
  - What is the cause of the discrepancy?
  - Specific staff group?
- CLTI should be referred urgently. Do you think primary care clinicians are aware of the urgency of these symptoms, and have the ability to act in a timely manner?
  - If not, what are they lacking?
  - What is the reason behind your answer?
- Do you think primary care clinicians have sufficient knowledge of the referral pathway for patients with suspected CLTI?
  - If not, what are they lacking?
  - Why is there such a discrepancy?
- Thinking about the [unit] pathway, do primary care clinicians have the skills to negotiate an urgent referral within the context of the pathway?
  - Do you think the [unit] pathway is easy to negotiate from primary care clinicians
  - If not, what are they lacking?
- Do primary care clinicians have an appreciation of the harms of failure to urgently refer?
  - If not, what are they lacking?
  - What prevents them from recognising the harms?
- We’ve spoken about clinicians, how about patients from the first symptom?
  - Do patients recognise symptoms?
  - Can they access the care they need?

Further questions

- Now, we’ll look at the process map that we created together with you and other members of the team
- This is the process map put together by the vascular unit you work at. Is it accurate? You will maybe only be able to tell me about your bit of the pathway – that’s fine
  - What is good about your current referral pathway?
    - Why? Can you give me an example?
  - What specific areas of this referral pathway could be better and how?
    - Any others?
    - How would you ideally like to receive a referral?
      - Why?
      - Would a proforma help / Does your proforma help?
    - How would you ideally like a referral to be triaged?
      - Why?
    - How would you ideally like to see a patient with CLTI?
      - Why?
  - Your process map is heavy on the use of ___ (VSN / podiatry / vascular surgeon). What is good about this?
    - What sort of feedback is given by patients?
    - Are there any downsides?
- We’ll now talk about some of the delays in the system demonstrated on the map, and how they could be improved
- Delays
  - We’ve discussed some of the issues with primary care clinicians. What do you think are the most important contributors to delays in the process from patients developing a symptom and being assessed by your team?
    - Where in this referral process?
      - Why?
    - Is it different if they live near the hub or the spoke?
    - Referral set up across network into hub or spokes?
    - Is it different if they have any specific comorbidities? Like diabetes?
- Potential resources
  - How do you think we can improve delays in the system within current resource available?
  - What do you think would help reduce delays in care of patients with CLTI if resources weren’t an issue?
    - Why will this work?
  - Is there anything already in place in other specialties / locations that you think might help?
    - Why are they good?
    - Are there any downsides?
  - Have you heard of any suggestions at conferences or in newspapers that do you think wouldn’t help?
    - Why?
  - Does your unit engage with primary care clinicians?
    - Why / why not?
    - If yes, what has it been like?
- Now, we’re in fantasy land thinking about what would be ideal. This can be wider than just your specific area, so thinking about the entire process map and all patients that may need vascular surgery assessment
- Pathways
  - What would your ideal pathway for a patient from first symptom to assessment by vascular surgery look like?
  - We’ve said your unit uses ___ (VSN / vascular surgeon / podiatrist). Would expanding the roles of any other specialties help reduce delays to assessment?
    - Expanding podiatry role for non-diabetics?
  - This is a generic version of the process map showing all pathways used across the country. Are there any pathways you see on the map that might improve the process in your unit?

Thank you

- Thank you so much for your time
- Is there anything you’d like to add on the topics we’ve discussed?
- Do you have any questions?

Next steps
